# Immersive educational curriculum on intracoronary optical coherence tomography image analysis among naïve readers

**DOI:** 10.1186/s12909-022-03704-0

**Published:** 2022-10-12

**Authors:** Nicholas Kassis, Joseph R. Weber, William Adams, Lucas Burke, Matthew P. Laubham, Mark Pelka, Nkiru Osude, Matthew Schreier, Samuel Robertson, Emily Janak, John J. Lopez

**Affiliations:** 1 Department of Medicine, Stritch School of Medicine, Division of Cardiology, Loyola University Chicago, Loyola University Medical Center, 2160 S. First Ave, Maywood, IL 60153 USA; 2 Department of Internal Medicine, Cleveland Clinic Foundation, Cleveland, OH USA

**Keywords:** Optical coherence tomography, Education, Curriculum, Training, Image interpretation

## Abstract

**Background:**

Optical coherence tomography (OCT) is an intravascular imaging modality for analysing coronary vessels. Image interpretation remains an obstacle for novice readers due to technical artefacts and uncertainty in tissue characterization. Despite an expanding clinical and research role for OCT, few training efforts exist, and there is an absence of a national standardized educational curriculum. We sought to determine whether an interactive, feedback-based OCT curriculum improved image interpretation among naive readers.

**Methods:**

Naive OCT readers completed both a Standard curriculum, comprised of self-directed didactics and consensus statements, and an Augmented curriculum, which provided real-time digital feedback of feature identification and measurements. Modules were separated by a minimum one-week washout period. After each module, and blinded to the exam answers, subjects completed an identical expert-designed 413-item exam to assess technical knowledge and ability to identify and measure vessel features. Performances were compared using Exact Wilcoxon signed-rank tests.

**Results:**

Among the 7 included subjects were 3 medical students, 3 internal medicine residents, and 1 cardiovascular medicine fellow with no prior OCT experience. The technical knowledge score (maximum 13) was significantly higher with the Augmented compared with the Standard curriculum (median 11 vs. 7, *p* = 0.03). After undergoing the Augmented curriculum, all 7 subjects were able to identify features of plaque rupture (Standard curriculum: 5/7 subjects, *p* = 0.5) and macrophages (Standard curriculum: 6/7 subjects, *p* = 0.99), differentiate the components between red and white thrombus (Standard curriculum: 6/7 subjects, *p* = 0.99), and characterize lipid plaque by attenuation, signal, homogeneity, and borders (Standard curriculum: 5/7 subjects, *p* = 0.5). Performances on the remaining exam portions did not differ between curricula.

**Conclusions:**

The need for standardized, effective training in OCT image interpretation is increasingly essential as the intravascular imaging modality becomes widely utilized among interventional cardiologists and trainees. A novel interactive OCT curriculum enhanced naive readers’ technical knowledge and may supplement traditional self-learning in refining analytic skills.

## Background

Optical coherence tomography (OCT) has emerged as a core intravascular imaging modality for characterizing structural features of coronary vessels and atherosclerotic lesions and for optimizing coronary stent implantation in select patients [1–3]. Efficient, accurate interpretation and analysis of OCT images is of critical importance yet remains an obstacle, particularly for novice readers. A recent survey-based report found that despite a sizable portion of interventional cardiology fellows-in-training noting sufficient exposure, only 18% cite preparedness to independently perform and interpret OCT [4]. While the absence of a national educational directive for OCT training may be contributory, the challenge is partly rooted in technical features that create imaging artefacts such as tangential signal dropout (OCT image beam strikes the vessel tissue at an oblique angle) and superficial shadowing, leading to misclassification of coronary plaques [5]. Movement artefacts, poor flush quality (inadequate displacement of blood from the vessel lumen), and non-centred cross-sectional OCT fibres can further complicate image analysis [6]. Finally, nuances of interpretation such as differentiating calcium deposits and lipid pools as well as interpreting complex, mixed plaque features can be vexing [7]. Often these pitfalls are avoided only by highly experienced readers trained to resolve ambiguities and recognize interpretation biases through countless hours of trial and error. Despite an expanding clinical role for OCT, no standardized training curriculum or educational tool is currently widely available. We sought to augment traditional self-learning efforts with a deliberate feedback-based OCT reading curriculum designed for new readers to master the basics of image interpretation while gaining a fundamental understanding of its complex subtleties.

## Methods

Overall, 7 naïve OCT readers individually underwent both a self-directed 'Standard Curriculum' (SC) and an ‘Augmented Curriculum’ (AC). The SC was comprised of lecture series in the form of 3 comprehensive presentations previously used at national conferences, consensus guidelines [8], and instructional documents [9], reviewed over a period of two weeks. The interactive on-line AC provided real-time feedback and detailed explanations of feature and artefact identification, plaque characterization and measurements, coronary vessel basics, and stent assessment. Immediate feedback was provided to subjects in the form of written answers that included thorough content explanations and associated references, as well as via expert-annotated, freeze-framed cross-sectional images located in a separate folder within the imaging software. All subjects underwent the SC prior to the AC, with modules separated by a minimum one-week washout period. After each module, and blinded to the exam answers, subjects completed an identical 413-item exam designed by three experts to assess overall knowledge and ability to identify and measure coronary plaque and vessel features. The exam content spanned moving-images consisting of multiple tomographic ‘slices’ from 10 unique coronary vessel segments.

**Fig. 1:**
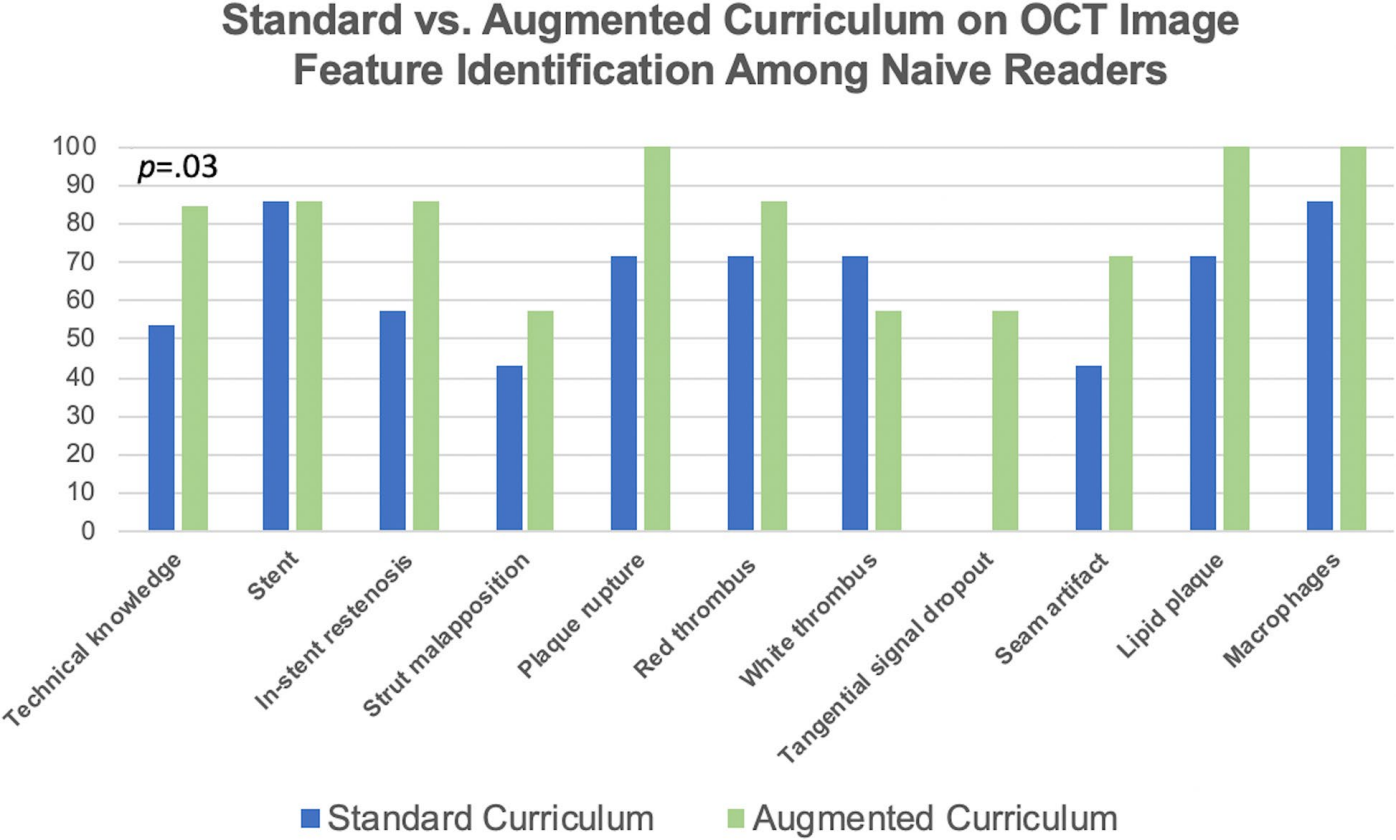
Impact of an interactive educational curriculum on optical coherence tomography (OCT) image analysis among naïve readers. Performances on OCT image feature identification after Standard versus Augmented Curriculums. Overall technical knowledge score (maximum 13) was significantly different between curricula, while performances on identifying all individual image features were similar

In order to best reconcile varied learning and test-taking skills, both quantitative and qualitative measures were assessed using multiple-choice questions, open-ended questions, and image-based feature identification (Fig. 1). Gold-standard answers were pre-determined by expert readers. For each continuous variable, a delta score was calculated as the difference between the gold standard value and participant entry. For categorical questions, the maximum possible score was 163 points, with 13 points attributed to a technical knowledge portion including identifying images of plaque features and artefacts. Exact Wilcoxon signed-rank tests were used to assess whether the distribution of delta scores changed after each curriculum, and whether performances on categorical features differed between curricula. The study protocol was approved by the Institutional Review Board, and informed consent was waived owing to the study design with anonymized data.

## Results

The cohort included 3 medical students, 3 internal medicine residents, and 1 cardiovascular medicine fellow, all with no prior OCT reading experience. Table 1 illustrates sample content from the AC with most content repeated across multiple OCT study segments. Table 2 details sample exam questions within pre-specified segments and frames of interest.

**Table 1:** Sample content from the ‘Augmented Curriculum’ for OCT image interpretation

Augmented Curriculum
**Basics, plaque type/features, and measurements**
1. Measure the outer area of the catheter sheath
2. Identify and outline the vessel lumen, intima, media, and adventitia
3. Measure the luminal and external elastic membrane areas
4. Identify the guidewire shadow by tracing its borders with the “length” tool
5. Measure the maximal intimal thickness three times to ensure consistency
6. Does this cross-sectional image classify as intimal thickening or fibrous plaque?
7. Adjust the calibration using the “adjust calibration” tool at the bottom of the screen
8. Identify the predominant plaque type
9. Measure the calcium and lipid areas and arcs
10. Measure the minimum fibrous cap thickness three times
11. Does this lipid plaque classify as a ThCFA or TCFA?
12. Identify and describe the plaque features and list possible etiologies of the observed patterns
**Stents**
1. Using the “area” tool, outline the stent circumference. Is this stent well-apposed?
2. What is the maximal distance from the stent to the vessel wall?
3. What is the predominant feature of this cross-sectional image?
**Artifacts**
1. Measure the luminal area
2. Identify the imaging artifacts
**Practice pullback**
1. Identify the reference and minimal lumen frames and measure the areas
2. Calculate the percentage area stenosis of the minimal lumen area
3. Identify the proximal and distal edges of the lesion by frame number
4. Calculate the lesion length

Sample questions from the ‘Augmented Curriculum’, many of which were repeated across multiple OCT pullback segments. Immediate feedback was provided to subjects via written answers that included detailed content explanations and associated references, as well as via expert-annotated, freeze-framed cross-sectional images located in a separate folder within the imaging software

*ThCFA* Thick-cap fibroatheroma, *TCFA* Thin-cap fibroatheroma

**Table 2:** Sample content from the expert-designed OCT image interpretation examination

Exam Content
1. Identify the minimal lumen frame, MLA, MLD, and percentage area stenosis
2. Identify the reference frame, reference lumen area, and reference mean diameter
3. Identify the location of all observed branches by frame number
4. Measure the minimal and mean FCT
5. Measure the total calcium and lipid arcs
6. Identify the presence of TCFA
7. Characterize the plaque type as fibrotic, calcified, lipid, mixed, or no plaque
8. Measure the length of TCFA as defined as < 65 µm
9. Measure the length of TCFA as defined as < 100 µm
10. Technical knowledge
*▪ Image-based free text*: identifying the OCT feature
*▪ Multiple-choice*: characterizing and identifying features of lipid plaque, plaque rupture, macrophages, white and red thrombus, and vessel wall layers

Questions 1–9 reflect content within pre-specified segments and frames of interest. The expert-designed exam included 413 content questions across 10 unique coronary vessel segments

*MLA* Minimal luminal area, *MLD* Minimal lumen diameter, *FCT* Fibrous cap thickness, *TCFA* Thin-cap fibroatheroma, *OCT* Optical coherence tomography

Technical knowledge scores were significantly higher with the AC relative to SC (median 11 vs. 7, *p* = 0.03). Specifically, 0/7 and 3/7 subjects identified tangential signal dropout and seam artefacts with the SC, respectively, compared with 4/7 and 5/7 with the AC, respectively (Fig. 1). After undergoing the AC, all 7 subjects accurately identified features of plaque rupture (SC: 5/7 subjects, *p* = 0.5) and macrophages (SC: 6/7 subjects, *p* = 0.99), differentiated the components between red and white thrombus (SC: 6/7 subjects, *p* = 0.99), and characterized lipid plaque by attenuation, signal, homogeneity, and borders (SC: 5/7 subjects, *p* = 0.5). Performances on the remaining exam portions, including continuous measurements of image features and identifying plaque type (SC: median 17/50 vs. AC: median 22/50 total points, *p* = 0.63) and TCFA (SC: median 40/50 vs. AC: median 36/50 total points, *p* = 0.99), did not differ between curricula.

## Discussion

Our study illustrates that a novel interactive OCT image reading curriculum enhances naïve readers’ technical knowledge and may supplement traditional self-learning in refining skills to analyse coronary vessel features. These findings portend key implications in the development of a standardised educational program for novice readers across all stages of medical training. Ultimately, a structured feedback-based curriculum may benefit those seeking to improve their capacity to interpret and apply intracoronary imaging to guide diagnostic and therapeutic decision-making in the cardiac catheterization laboratory. This is particularly important as the marked advantages of OCT become increasingly apparent commensurate with the growth of complex interventional cases and wider adoption of device technology [1, 10].

Despite a scarcity of educational and curricular data related to OCT in the interventional cardiology literature, our findings are consistent with those of prior reports in radiology [11] and instructional psychology [12] that support an interactive and case-based learning format, with special emphasis on implementation and real-time feedback via digital tools [11, 13, 14].

Failure to demonstrate an improvement in the accuracy of quantitative measurements following the AC may represent the small sample size or an inherent limitation in the study design. No contact was made between exam creators and participants regarding exam content in order to minimize bias. Therefore, key measurements were subject to participant interpretation given the lack of consensus in characterizing plaque type (varying minimum arc / number of quadrants in *Cartesian coordinates*) and defining thin-cap fibroatheroma (varying minimal fibrous cap thickness threshold) [15]. Resolving these ambiguities will require sufficient education and updates to consensus standards, which were written prior to much of the current work in OCT clinical research [8]. The goal of our study was not to create experts from naïve readers, but rather to enhance and expedite their training to become capable readers. Expert oversight and routine experience are crucial to becoming proficient. We further acknowledge the potential limitation of a pre-post study design with subjects repeating the identical exam after the washout period between curricula; we attempted to mitigate this by blinding the subjects to the initial exam answers.

As the role of OCT expands in both the clinical and research arenas, and OCT training remains limited to national conferences or informal observation, the need for accessible and effective standardized education of new readers is increasingly important. Albeit a small sample of trainees, our in-depth analysis demonstrates for the first time that novice OCT readers may benefit from a practical interactive curriculum, one that stands to further improve with additional iterations. The methodology, data, and curriculum and exam contents in their entirety are available to any researcher upon request, for the purpose of conducting similar analyses.

## Conclusions

In view of the increasing need for standardized training in OCT image interpretation among naïve readers, a novel interactive OCT curriculum enhances technical knowledge and may supplement traditional self-learning in refining analytic skills.

## Data Availability

The datasets used and/or analysed during the current study are available from the corresponding author on reasonable request.

## Abbreviations

OCT: Optical coherence tomography
SC: Standard Curriculum
AC: Augmented Curriculum
